# Dyspepsia—Its Treatment

**Published:** 1903-08

**Authors:** Walter U. Kennedy


### Abstracts and Selections.

### Dyspepsia—its Treatment.

BY WALTER U. KENNEDY, M. D.

Before discussing the treatment of dyspepsia, it is proper to allude to its pathology and symptoms within the limits of its usual etiology. It is sometimes associated with serious organic diseases, when relief can only be reached by addressing our treatment to those organic diseases of which it is a logical sequence.

In discussing the treatment here, it is our purpose to regard dyspepsia simply as a derangement in the natural process of digesting and assimilating food, and more especially a faulty performance of the functions of the stomach. Although it is a medical topic which, from time immemorial, has engaged the attention of layman and doctor, the suffering which it has entailed, and its universal prevalence in civilized life, entitle it to the careful study of every member of the medical guild. The most prominent symptoms of dyspepsia are, want of appetite, nausea, vomiting, flatulence, gastrodynia and pyrosis. Associated with it we often find constipation and diarrhea. The former is usually induced or aggravated by an atonic or torpid condition of the intestinal musculature, the latter by an irritable state of the intestinal mucosa. Of course, improper diet and faulty hygiene are potent additional factors in the development of these incurrent complications. Intemperate indulgence in eating or drinking, especially at short and irregular intervals, with the consequent alterations in the gastric solvents, are frequent provocations of disordered digestion. The gastric juice may be in excess, deficient or completely suppressed, or it may be abnormal in quality.

These changes in quality may represent an excess or deficiency in hydrochloric acid or pepsin, with resultant decomposition and fermentation of food and flatulent distension, accompanied by an endless chain of morbid phenomena. To meet such conditions there are three procedures which demand paramount attention—diet, hygiene and artificial digestants.

With regard to diet, we can say it must be adapted in quality and quantity to the disordered conditions of the stomach. In some cases it is necessary to be particular as to elements of food which are selected. If there is a tendency to acid fermentation, starchy substances must not be given. Under the head of hygienic management, many things must be considered, among which is a due amount of exercise at the proper time, not immediately before or after a meal, avoidance of mental work and worry, temperance in all things, promotion of the action of the skin by means of the bath and warm clothing, and a careful composure of all the mental faculties.

With regard to artificial digestants, numerous medicinal agents have been employed with more or less success. In the use of these agents a discriminating judgment is necessary. Some of them will answer the purpose very well in acid conditions and some are only active in alkaline or neutral conditions of the stomach; but can we not find a digestant that is active in all of these conditions, and at the same time possessed of powerful antiseptic and tonic properties?

I believe that Caripeptic Liquid (Upjohn) will more generally meet all of these requirements than any other medicinal agent. It contains all the proteolytic ferments of the Cariaca Papaya tree, and as a digestant is much more active than pepsin, dissolving fibrin readily in acid, neutral or alkaline liquids. It is not only an efficient remedy in all forms of functional dyspepsia, but is palatable and pleasant, and this is a strong recommendation for any drug which is extensively employed in the treatment of disease.

I have used it freely with a large number of dyspeptic patients, obtaining uniformly favorable results. The following cases, taken from my case book, show some of the therapeutic results of its employment in my practice:

Case 1.—February 25, 1903. Miss J. T., age 30, complained of anorexia, nausea, flatulence, gastrodynia, pyrosis, belching and distension of the stomach; suffers most when stomach is empty, bowels constipated, tongue coated, breath offensive. Prescribed Pil. Caripeptic Comp., two pills at bedtime and one in the morning. Milk diet. Two teaspoonfuls of Caripeptic Liquid after each supply of milk. Gentle exercise and warm bath. February 27th: Stomach improved; some appetite. Ordered small allowance of vegetables and fruit, with a little mutton. Continued Caripeptic Liquid. March 2nd: Patient very much improved; no disagreeable symptoms. Continued treatment for another week, when patient was discharged.

Case 2.—February 28, 1903. H. J., male, age 35. Had long been in the habit of great excesses in diet and free use of spirits. Had lately suffered from an attack of la grippe. Complained of pain in epigastrium, nausea and vomiting. Had no appetite; tongue red and coated; burning sensation in stomach; eructation of a thin, watery fluid of a sourish taste; some diarrhea. Prescribed small doses of calomel with bicarbonate of soda, to be followed by subnitrate of bismuth. Milk and vegetable diet, with small allowance of tender beefsteak. No alcoholic stimulants. Dessertspoonful of Caripeptic Liquid, after each meal and at bedtime. March 2nd: Patient improved; stomach retains and digests moderate quantities of food without any discomfort. Treatment continued. March 6th: Patient improving rapidly. Increase and diversify diet. Continue Caripeptic Liquid. March 13th: Patient discharged, and resumed his ordinary occupation of teamster.

Case 3.—March 4th. Isaac R., age 29. Occupation, traveling salesman. Pale, purple lips, pulse 80, quick, irregular; fullness in region of stomach, flatulency, epigastric pain, no appetite, costive, more uncomfortable when stomach is empty. Habits irregular, especially with regard to eating and sleeping. Ordered rest and light nourishing diet. Prescribed Pil. Caripeptic Comp., two at bed-time and one in the morning, followed with dessertspoonful of Caripeptic Liquid, after each meal and at bed-time. Continued this treatment for two weeks, when patient returned to his usual occupation entirely relieved.

Case 4.—March 10th. W. H., male, age 40, printer. Came with complaint of flatulence, and distension of stomach after eating, eructations, languor, listlessness and occasionally vertigo. Diet—milk and peptonized beef tea. Prescribed dessertspoonful of Caripeptic Liquid after each meal and at bed-time. March 15th: Patiently rapidly improved, continued treatment. Gradually changed and increased food. A week later the patient resumed his daily work, entirely relieved of every symptom.

